# Access to high-cost drugs for advanced breast cancer in Latin America, particularly trastuzumab

**DOI:** 10.3332/ecancer.2019.898

**Published:** 2019-01-22

**Authors:** Carlos Henrique Barrios, Tomás Reinert, Gustavo Werutsky

**Affiliations:** Latin American Cooperative Oncology Group, 99 A, Av Ipiranga 6681, Porto Alegre, RS 90619-900, Brazil

**Keywords:** breast neoplasm, HER2, trastuzumab, drug therapy

## Abstract

Provision of high-level healthcare is a challenge for all low- to middle-income countries (LMICs) since healthcare systems are heterogeneous, face many challenges such as inadequate funding, inequitable distribution of resources and services and usually are not adequately equipped to deal with a huge problem such as breast cancer. The development of anti-HER2 therapies can be considered one of the most important examples of the translation of molecular biology knowledge into clinical benefits for cancer patients. While a variety of novel therapeutic strategies are emerging, current treatment regimens remain focussed on targeted therapy with monoclonal antibodies, mainly trastuzumab, the first agent developed in this field. While these results have revolutionised the outcome of HER2+ patients in clinical trials and in high-income countries where they are widely available, results have not impacted the natural history of this aggressive disease in most of the world. Unfortunately, the availability of these drugs is far from universal in many LMICs, and in Latin America, in particular, patients with HER2+ breast cancer are treated exclusively with standard chemotherapy, a more toxic and less efficient therapy. While the complexity of the situation and the multiple factors that have an impact in this scenario are recognised, we need to map the future and develop feasible strategies to address possible solutions to the problem of drug access. A clear and unbiased diagnosis of the situation is a good starting point. Defining healthcare priorities and a clear strategy for the allocation of resources is difficult but mandatory. In this article, we will discuss current and future challenges regarding access (and lack of access) to high-cost cancer drugs in Latin America, with a focus on anti-HER2 therapies.

## The global burden of cancer

Cancer represents a significant and growing global concern. With the progressive control of other causes of mortality and the increase in life expectancy, cancer is a truly major universal health challenge. It is projected that new cases of cancer will increase from about 14 million in 2012 to 22 million in 2030, with most cases in low- to middle-income countries (LMICs) located in Africa, Asia and Latin America [1]. These regions are facing an epidemiologic transition to a pattern of morbidity and mortality similar to high-income countries (HICs), with a rising incidence of certain forms of cancer such as colon, lung, prostate and breast cancer [2].

According to the GLOBOCAN database, breast cancer is the most frequent cancer among women in the world, the most frequent cause of cancer death in women in less developed regions and the second most frequent cause of cancer death in more developed regions [3]. It is noteworthy that breast cancer incidence rates are increasing in most countries, but mortality rates are decreasing only in HIC [4].

Recently reported data from the United States indicates a 26% decrease in overall cancer mortality over the past two decades with breast cancer deaths reducing an impressive 39% from 1991 to 2015 [1]. While advances in screening, early detection and adjuvant treatment are mostly responsible for the decline in mortality in developed countries, most new cases are recorded in developing regions where death rates are increasing and early detection efforts are insufficient with a most diagnosis of tumours that are locally advanced or metastatic [4].

Currently, provision of high-level healthcare is a challenge for every country in Latin America, since health ministry’s policies and healthcare systems are heterogeneous, face many challenges and usually are not adequately equipped and unprepared to deal with problems such as cancer. Within this context, our objective in this paper is to explore the issue of access to high-cost cancer medications in Latin America, with special focus in HER2-positive (HER2+) breast cancer and access to anti-HER2 therapies.

## Current scenario of breast cancer care in Latin America

Breast cancer is the most common cancer in Latin American women, and, in most cases, it is diagnosed at later stages. Due to demographic transition, the breast cancer rate will approach epidemic proportions with great social and economic consequences [5]. Therefore, we expect that the disease will have a substantial social and economic impact in most societies in the forthcoming decades. As a practical consequence to these figures, we need to recognise the clearly documented “perverse” relationship between gross domestic product (GDP) and trends in the breast cancer incidence and mortality/incidence ratio. The higher the GDP is, the higher is the incidence of breast cancer with a lower mortality/incidence ratio whereas in countries with lower GDP there is a much higher mortality/incidence ratio [6]. Therefore, the burden of breast cancer mortality lies in the developing world, where approximately 70% of such deaths occur [7]. Survival at 5 years varies from around 80% or more in HIC to 60% in middle-income countries and 40% in low-income countries [8]. For the Latin America and Caribbean regions, an estimated 1.7 million annual cases of cancer will be diagnosed by 2030, and more than 1 million cancer-related deaths will occur annually [1].

Latin America and the Caribbean have a heterogeneous population with distinct cultural, ethnic and socioeconomic backgrounds and provision of adequate care for breast cancer patients remains a major challenge. The incidence of cancer is 63/100.000 in the region, much lower than that seen in the US (300/100.000) and Europe (264/100.000). However, a patient that develops cancer in Latin America and the Caribbean has a much greater chance of dying from the disease. More advanced stages at presentation and difficulties in accessing optimal treatment care are the main reasons for this disparity. While in the US more than 60% of patients are detected in earlier stages of the disease, with much higher curative possibilities, data from Brazil and Mexico indicate that more than 60%–70% of cases are diagnosed in more advanced stages requiring more complex management with lower chances of cure [2, 9].

The rising cancer incidence is closely linked to the increasing economic burden of cancer, alongside with the growing investment needed for disease prevention, diagnosis and treatment. While it is estimated that new cancer drugs have accounted for 50–60% of the increase in cancer survival rates since 1975, at the same time, they are also associated with a significant increase in costs [10]. In Europe, total cancer drug sales more than doubled between 2005 and 2014, increasing from €8.0 billion to €19.8 billion [10]. The increasing costs are related to the rising prices of newly approved agents, the growing number of cancer patients requiring treatment, and superior treatment efficacy leading to increased survival rates and subsequent higher cumulative treatment costs over a patient’s lifetime.

Taking into consideration where new drugs are consumed globally, we identify an awkward discrepancy that clearly helps to pinpoint some of the challenges we will face ahead. One of the most important aspects in reducing cancer mortality is the availability and health system uptake of innovations and life-saving cancer drugs [11]. Analysing where new drugs are consumed in the world, we identify an awkward discrepancy that clearly helps to pinpoint some of the challenges we will face ahead. Among the medications that have been released to the market over the past five years, 64% are sold exclusively in the US, 24% in Western Europe and 7% in Japan. This leaves only about 5% for the rest of the world. It should not be a surprise that healthcare outcomes clearly correlate with these numbers [6, 12]. As expected, lack of availability and access to anti-HER2 medications has an important impact on outcomes [6, 13].

Universal healthcare coverage is not the rule in Latin American countries, and even in those regions where the entitlement to oncology services is guaranteed by law, it is not accompanied by the necessary resources. Vast disparities in access to breast cancer care in Latin American countries, and even within the same country, exist and lead to unequal outcomes. All these factors are compounded by the fact that in Latin America and the Caribbean each country has unique healthcare system issues. In the region, we identify inconsistent or absent health care planning addressing cancer control associated with lack of resources to finance public healthcare. As a result, fragmented systems with significant discrepancies in care in different regions within the same country are frequently the case with unplanned and unfair distribution of the scarce resources generally spent in the absence of a clear or strategically defined plan. Infrastructure needed for cancer care and processes of care suffer accordingly, leading to worse outcomes and poor or highly inadequate outcomes. While in most countries we can recognise inequitable healthcare systems, some initiatives of universal healthcare systems have been launched such as in Mexico or Brazil [9].

Further challenges include a clearly unbalanced distribution of healthcare specialists and facilities, generally concentrated in urban centres and most populated areas. While having availability where most of the population lives is appropriate, this still leaves a large proportion of the rural population unassisted [2]. These healthcare issues are not exclusive to Latin American and the Caribbean and can be clearly identified in the analysis of other regions of the world in different LMICs [14, 15].

## Contemporary management of HER2+ advanced breast cancer

The significant achievements that led to the reduction in breast cancer mortality observed in developed societies clearly demonstrate that it is possible to curb the devastating impact of this disease. Recent reports from Canada indicate that important improvements have been made in the management of early stage breast cancer with a clear decrease in the initial peak of recurrence when comparing results from the past two decades. These benefits were seen in all sub-types of the disease and especially in HER2+ and triple-negative tumours [16]. Furthermore, French investigators recently presented real-world data (RWD) on the management of women with advanced breast cancer and showed that the survival rates have improved impressively for the HER2+ group of patients, with contemporary survival rates higher than 50 months. It is noteworthy that results in patients with luminal and triple negative tumours have not improved at the same rate, indicating a clear unmet need for the development of new therapeutic strategies for these patients [6, 17]. Notably, it is important to mention that these results come from a country where access to the majority of new drugs is available for most patients and other important aspects with impact on breast cancer mortality, such as screening and early diagnosis programmes, are dealt with by appropriate and effective strategies.

Breast cancer is a heterogeneous disease with many distinct biological sub-types. Approximately 15–25% of breast cancers are classified as HER2+, a sub-group of tumours with a more aggressive clinical phenotype and worse prognosis due to unregulated cell growth mediated by overexpression of the HER2 protein [18]. However, patients with HER2+ breast cancer are sensitive to and derive significant clinical benefits from treatment with anti-HER2 agents. Currently, there are multiple effective therapies for HER2+ breast cancer, all of which block the HER2 pathway at different levels (intra or extracellularly). Since the approval of the first HER2-targeted therapy, trastuzumab, other anti-HER2 targeted agents have been developed and tested in the metastatic and in the (neo)adjuvant settings, including lapatinib, pertuzumab, neratinib and TDM1 [19]. These agents have been associated with significant clinical benefits including substantial increases both in response rates and in overall survival both in early stage and in metastatic disease.

The development of anti-HER2 therapies can be considered one of the most important examples of the translation of molecular biology knowledge into clinical benefits for cancer patients. A variety of novel therapeutic strategies are emerging, but current treatment regimens remain focussed on targeted therapy with monoclonal antibodies, particularly trastuzumab, the first agent developed in this field.

Trastuzumab was approved in 1998 in the USA and a couple of years later in the European Union for patients with metastatic disease. A pivotal study demonstrated a significant prolongation in overall survival when added to standard chemotherapy agents in patients with metastatic disease [20]. This early success led to the deployment of a number of large adjuvant trials trying to demonstrate a benefit with the early administration of trastuzumab. The initial reports of these studies in 2005 were uniformly positive, demonstrating longer disease-free survival and later, overall survival with the introduction of adjuvant trastuzumab [21]. Regulatory approval for use in the adjuvant setting was granted in most countries after 2006.

Demonstrating benefits in the advanced and early disease settings, trastuzumab was also shown to improve pathological complete response rates when used in the neo-adjuvant setting, covering as expected, the whole spectrum of presentations of the disease [22]. Currently, the American Society of Clinical Oncology (ASCO), the National Comprehensive Cancer Network and the European Society of Medical Oncology (ESMO) as well as most other treatment guidelines for breast cancer recommend the addition of anti-HER2 agents to systemic chemotherapy or endocrine therapy for the treatment of patients with early stage, locally advanced and metastatic HER2+ breast cancer. Furthermore, trastuzumab was included in the World Health Organisation (WHO) Essential Medicines List. This is an important international achievement as it officially defines this anti-HER2 drug as critical for the management and improvement of cure rates in early and locally advanced stages of breast cancer patients with this particular sub-type.

It is important to point out that after trastuzumab, other efforts led to important advances in anti-HER2 therapy and other innovative medications became available, adding to the results in the management of these groups of patients [23]. The combinations of these different agents with available chemotherapies and the possibilities of sequencing as a consequence of having them available have led to unprecedented results in the survival of patients with HER2+ advanced breast cancer. The CLEOPATRA trial tested double blockade with trastuzumab and pertuzumab versus single trastuzumab both in combination with standard taxane-based chemotherapy in patients with metastatic disease, and demonstrated a median overall survival in excess of 54 months [24], unprecedented results that were confirmed in the RWD reported by French investigators as mentioned above [25].

While these results have revolutionised the outcome of metastatic HER2+ patients in clinical trials and in HIC where they are widely available, results have not impacted the natural history of this aggressive disease in most of the world. Unfortunately, the availability of these drugs is far from universal and today, in many regions and in Latin America in particular, patients with HER2+ disease are still treated exclusively with chemotherapy. We will address some of the data available in this regard in our region.

## Access and lack of access to trastuzumab

As reported in different regions, barriers to optimal access to trastuzumab are multi-factorial and include issues related to drug cost and high overall treatment expenses for patients in ill-equipped health systems [26, 27]. With the rising cost of new therapeutic strategies for cancer care, in order to improve and provide affordable treatment, it is imperative to concentrate on medical interventions that are clinically impactful in real life and not only statistically significant in the research setting with a marginal clinical benefit [6]. In this regard, ESMO and ASCO have proposed criteria to ascribe value to the improvement that different experimental treatments bring to patient outcomes when compared to standard therapies [26]. Of note, we need to acknowledge that we frequently accept questionable statistical differences as sufficient to incorporate new drugs. While recognising the incremental improvement in outcome results that has characterised cancer research over the past few decades, it is important to be critical of what can be statistically significant but clinically irrelevant.

Moreover, one of the most challenging aspects of drug development is the translation of the results obtained in clinical trials to the clinical practise scenario. While a clinical trial is a coordinated experiment that has very tightly controlled inclusion and exclusion criteria and precise pre-defined procedures for most if not all predicted situations, clinical practise does not fit and does not follow the same setup. Reproduction of any observed benefit in a real-world patient scenario is arguably the most important step in the impact of any new development. If a result cannot be reproduced in a real population care situation it becomes of questionable value. Generation of RWD is, therefore, an important endeavour that has been progressively singled out by the Food and Drug Administration (FDA) as key in the incorporation of new treatment alternatives, emphasising the fact that having an impact on the whole population remains the foremost objective [6].

Taking the example of anti-HER2 therapy development and trastuzumab, in particular, unquestionably we observe major improvements in patient care with categorical and reproducible consequences in outcomes. While trastuzumab is approved in most countries in the Latin American region, access to the drug is far from universal. While this may be considered a problem of LMICs, in reality, limitations in access have been documented in HICs as well [14, 28]. However, the impact of lack of access has been poorly analysed in the literature. One particular example coming from Brazil expands this argument.

Brazilian regulatory authorities approved trastuzumab following the demonstration of its benefits in the metastatic and the adjuvant settings (2000 and 2006, respectively). However, this regulatory approval does not guarantee universal access to the drug and mainly applies to the privately insured patients that represent approximately 20% of the country’s patient population. Women in the public health system, representing 80% of the population, remained with no access to trastuzumab in the adjuvant situation until early 2013 and until 2017 for metastatic disease. Table 1 describes the timeline of anti-HER2 agents’ approval [by the Brazilian regulatory agency Agência Nacional de Vigilância Sanitária (ANVISA)] and availability in the public health system (SUS). While the interval between FDA and ANVISA’s approval is relatively short, the gap between the approval of the drug in Brazil and availability in the public health setting is very long and in the case of drugs such as lapatinib and TDM1 that have been approved and used in the private setting for many years but remains unavailable for patients treated in the public health setting [7, 9, 13].

Access to anti-HER therapy is historically very restricted in the Brazilian public health system. In 2006, only 5.6% of patients with HER2+ tumours received trastuzumab in the public healthcare system compared with 56% in the private sector [29]. With the underlying hypothesis that the consequences of lack of access can be estimated we appraised the potential number of deaths in Brazilian women with HER2-positive breast cancer as a consequence of the lack of access to the drug in this potentially curable clinical situation of early breast cancer. We estimated that close to 5,000 women died in Brazil with potentially curable early HER2 + breast cancer as a consequence of the lack of access of trastuzumab from 2006 to 2013 [30].

A similar analysis was performed by our group addressing the potential consequences of the lack of access to the combination of double blockade with trastuzumab and pertuzumab as utilised in the CLEOPATRA trial. We projected more than 700 early deaths of patients with metastatic HER2-positive metastatic breast cancer due to lack of access to optimal treatment [13]. Not surprisingly, RWD of survival for HER2 positive advanced breast cancer in Brazil has been estimated at 23 months while it is in excess of 50 months in the data presented from France where the treatment is available to all patients with the indication [25].

## Barriers to the incorporation of new therapies for advanced breast cancer

In some countries, the increasing economic burden is compromising access to new developments. At the same time, this places physicians under significant pressure to balance the cost of cancer care during each testing and/or prescribing decision. This can lead to patients being denied access to effective treatments. As discussed in Brazil, trastuzumab is not generally available in public settings for the treatment of metastatic disease. It is estimated that of 2,008 women diagnosed with advanced HER2-positive breast cancer in 2016, only 808 would be alive by 2018 if they receive only chemotherapy (the treatment offered by the public health system). Having access to the combination of chemotherapy and anti-HER2 therapy, an estimated 1,408 women would be alive in the same period instead [13].

Breast cancer health disparities also exist at a worldwide level, and the discrepancies between socioeconomic status, breast cancer presentation and outcomes have been well established. There is lack of RWD to provide decision makers with a credible information to confirm whether the results of randomised clinical trials are applicable to real-world clinical practise, or to understand how and why they differ [14].

Several studies demonstrate that not all patients with HER2+ breast cancer are treated with anti-HER2 therapy. In some cases, this reflects patient comorbidities and treatment preference. However, physicians worldwide encounter various barriers to prescribing biologic agents that may also contribute to underuse of anti-HER2 therapy [12] Age, socioeconomic and racial disparities have been demonstrated even in rich countries. A study published in 2015 reported that approximately 50% of patients aged 65 years and older do not receive neoadjuvant or adjuvant treatment with trastuzumab in the USA, especially those with a lower financial income. Furthermore, also in the US, black women were 25% less likely than white women to receive trastuzumab-based therapy within 1 year of diagnosis [31].

Similar disparities are also seen in LMICs in different parts of the world. In a RWD study from China, patients with early stage breast cancer in resource-abundant regions were more likely to receive trastuzumab than those in resource-limited regions (37% versus 13%, *p* < 0.05) [41]. Similarly, an observational study of Chinese patients with HER2+ tumours reported that 27% of those with advanced disease did not receive trastuzumab at any time after diagnosis and 49% did not receive trastuzumab in the first-line setting [14].

An international physician survey showed that 94% of responders from low-income countries and 63% from middle-income countries cited drug costs as a barrier to prescribing adjuvant trastuzumab [32]. In a survey with oncologists in the USA and emerging markets (Brazil, Turkey, Mexico and Russia), 37%–49% of respondents who reported not frequently prescribing trastuzumab cited lack of insurance coverage, and 37%–44% cited unavailability of the drug as common barriers to using anti-HER2 therapy across all clinical settings [33]. Furthermore, issues related to treatment costs, drug funding and reimbursement also led to delays or cancellation of treatment with trastuzumab.

In a 2011 international physician survey, the vast majority (92%) of respondents indicated that they routinely recommend 1 year of adjuvant trastuzumab; however, 47% reported having at least one case within the prior year in which anti-HER2 therapy was recommended but could not be started. Failure to begin the recommended treatment with trastuzumab was more frequently reported in LMICs (75%) than HICs (40%; *p* = 0.005) and most often cited by physicians from Africa (100%), Asia (89%) and Latin America (80%) [34]. Arguably, the 40% reported in HICs is an unexpected and surprising number.

Unquestionably, the economic impact on the health system is one of the main reasons for the lack of access to trastuzumab. Despite being considered an essential medicine by WHO and the established clinical benefit in early breast cancer, some studies have not found adjuvant trastuzumab to be cost effective in several Latin American countries [47]. In Colombia, a model of economic evaluation showed that the utilisation of adjuvant trastuzumab can prolong 0.8 quality-adjusted life years (QALY) compared with standard chemotherapy at an incremental cost-effectiveness ratio of US$71.491 per QALY gained [35]. In Brazil, it was showed to be cost effective in the private health insurance segment which covers only 20% of the population [29]. The evaluation of the cost effectiveness of interventions to prioritise resource allocation is critical to make innovative cancer drugs available and affordable in developing countries. A study evaluating the potential implications of trastuzumab global pricing policies in seven Latin American countries showed that in 2015 the medication was considered not cost effective [36].

In LMICs, the cost of cancer treatment frequently exceeds the average per capita income by many multiples. For example, the cost of a typical trastuzumab course, prescribed during the treatment of advanced breast cancer, is approximately 15 times the per capita monthly income of an average Indian [27]. Similarly, in Peru, therapy with trastuzumab costs more than three times the GDP per capita per disability-adjusted life year and cannot be considered cost effective [37].

A number of added issues should be taken into consideration. Regulatory aspects of drug approval are one of them. As an example, the Brazilian healthcare system is complex and harbours severe inequity issues leading to disparities in access to novel expensive therapeutic agents, especially in the public health system that, despite their scientifically confirmed clinical benefits, are not available to the vast majority of the population [9]. Keeping the balance between efficacy, cost and ethical availability of state-of-the art drugs is a significant problem in a country that has a long history of inequity and faces one of its worst financial crises ever. The incorporation of novel therapies must be assessed regarding its positive clinical benefit on public healthcare patients and the financial burden that its prescription would impose on the public health budget. This is a multifaceted discussion and several stakeholders are involved in the process—from health authorities to pharmaceutical companies, private health care providers, medical societies, researchers and civil society.

In the Brazilian public health system, access to innovative and high-cost drugs is low and, in general, is lagging many years behind in relation to the date of drug approval in developed countries. In addition, there is usually a similarly long timeframe from the drug approval and availability of new agents in the private setting until its availability in the public health system. It is important to mention that in reality huge disparities exist, even within public hospitals and probably the majority of patients treated for breast cancer outside major urban centres do not receive trastuzumab in the same proportion as patients treated in reference centres located in the major cities. Recently, expanding on this discrepancy, the Brazilian Clinical Oncology Society reported that there were 37 therapeutic indications there were available on private but not on the public health system [38].

Delays in drug approvals have been significant and this has measurable impacts in the outcomes of patients that are unlucky enough to face the diagnosis in between periods when the scientific evidence is already available but the drug is yet to receive regulatory approval. Submission requirements, lack of transparency on the evaluation process and delays in the submission of regulatory documentation by the pharmaceutical companies have also been cited as contributing elements for the delay in access.

## Moving forward

While the complexity of the situation and the multiple factors that have an impact in this scenario are recognised, we need to map the future and develop feasible strategies to address possible solutions to the problem of drug access. A clear and unbiased diagnosis of the situation is a good starting point. Defining healthcare priorities and a clear strategy for allocation of resources is difficult but mandatory. The hard decision of who will get what and when is the most important one. Well-designed comprehensive cancer plans adjusted to each country’s reality are mandatory and clearly the initial point in this discussion.

Better control and more appropriate strategic regulation of the drug development process is critical as the costs and time associated with the current model do not fit with the urgent needs our patient have for better results. This would be one of the many aspects that could introduce more pressure and transparency in the drug pricing process. More discussion of the price of drugs is obviously not the only one but one of the many elephants in the room.

Biosimilars could improve patient access to anti-HER2 therapies worldwide [39]. Biosimilars are biologic products that are highly similar to a licenced biologic (i.e. the reference or originator product), notwithstanding minor differences in clinically inactive components. Furthermore, they have no clinically meaningful differences in safety, purity or potency compared with the reference product [40]. Biosimilars have the potential to enhance accessibility to cancer treatments for patients and provide alternatives for decision makers such as prescribers, regulators, payers and policymakers [40]. The field of anti-HER2 therapy with antibodies as their most important players, is of particular interest in the discussion of biosimilar development [27].

Patents for several biologic drugs, including trastuzumab, have recently expired or will soon expire, which has stimulated the development of biosimilars [41]. Several countries have implemented abbreviated pathways for approval of biosimilars. To demonstrate biosimilarity, the WHO recommends conducting comparability and characterisation studies on biologic activity, physicochemical properties, process- and product-related impurities, and product stability. In addition, non-clinical studies on pharmacokinetics, pharmacodynamics, efficacy and safety properties are required [42]. Key points of various regulatory guidelines on biosimilar development have been comprehensively reviewed elsewhere [40, 42, 43]. Biosimilars have been integrated into clinical practise in the European Union for almost a decade. In 2006, somatotropin became the first biosimilar to be approved by the European Medicines Agency [44]. In 2015, a biosimilar of filgrastim became the first biosimilar to be approved by the FDA. Nevertheless, challenges to their effective use remain including lack of awareness and possibly acceptance among healthcare professionals, issues about clinical trials design and extrapolation of indications, and even potential political barriers [40].

In 2013, the trastuzumab biosimilar Hertraz (MYL-14010) was approved in India. In 2014, the trastuzumab biosimilar CT-P6 was approved in Korea for the treatment of early and metastatic HER2-positive breast cancer. A variety of other trastuzumab biosimilars are in various stages of development with phase III trials publication expected for the near future [45]. Recently, the trastuzumab biosimilar MYL-14010 was evaluated in a phase III clinical trial that compared MYL-14010 with Herceptin in terms of safety and efficacy. With 500 patients included in this trial, no significant differences were seen in terms of overall response rate and safety. These results were presented at the ASCO Annual Meeting and suggest that the trastuzumab biosimilar MYL-14010 could be a new treatment option for HER2-positive metastatic breast cancer [46].

In addition, modern and more transparent processes of regulatory drug evaluation and approval will certainly bring to light many of the local issues that generally play a significant role but remain hidden in bureaucracy. Often, undisclosed restrictions with questionable interpretations of scientific data conceal lack of resources, ill-designed allocation policies or other commercial issues that end up protecting individuals or committee’s decisions with negative consequences for cancer outcomes. More transparency would certainly help in the process and would identify the real problems easing the process of reaching a solution. At the end of the day, putting a price on how much each society is willing to invest or can invest in a particular disease scenario or patient is the main question we keep dancing around.

Clinical research initiatives represent a particularly important aspect of the discussion. While not a solution for the central problem, research participation, with all its different caveats, is a very easy and practical solution to access and at the same time helps to speed development, access possibilities and lower the cost of drug development. Short and rapidly recruiting trials are clearly less expensive than those with long and slow accrual. Tailoring research questions to the main problems faced by different populations and regions is certainly one major strategy to increase physician and patient participation in the process. Physicians and patients in LMICs may choose to participate in clinical trials as a means of accessing medications that would otherwise not be covered in health systems with limited resources. Ten years ago, 94% of oncologists practicing in Latin America reported that there was insufficient clinical and epidemiological research on breast cancer in their countries [47]. Important barriers to research were insufficient financial support (79%) and lack of dedicated time (62%) [6].

Addressing the development and stimulating clinical research in LMICs will certainly result in a very positive impact in cancer care. Latin American countries should develop strategies to foster local research aimed at the specific health problems of its population. Tailored research has the potential to develop more effective medicines adapted to local problems and to reduce production costs. Consequently, all these strategies could enhance the effectiveness of a health system while maintaining its affordability [48].

It is of paramount importance to note that, while this article has focussed on access to trastuzumab for advanced breast cancer, effective cancer control strategies have to be culturally appropriate and are context dependent. Comprehensive efforts should be able to involve data gathering, health education, prevention, screening, early detection, multidisciplinary care strategies and surgical and radiotherapeutic treatments in addition to access to anticancer drugs [28].

## Conclusion

Breast cancer is the most common cancer and it kills more women than any other tumour type in Latin America. Important disparities in the access of anti-HER2 therapies for breast cancer have been demonstrated in LMICs and are linked to inferior cancer outcomes. Consequently, every year thousands of women die as a consequence of lack of access to standard of care treatment. As expected, there is not a single generalizable solution. The likely answers to increase access to anti-HER2 therapies for Latin American patients and decrease the existing disparities are usually complex, context dependent and depend on an intense interaction and active participation of different players requiring a passionate involvement of engaged local experts.

## Funding

None.

## Conflicts of interest

Dr Barrios´ disclosures:

- Honorarium, consulting/advisory role, research funding and meeting support from Novartis, Roche/Genentech, Pfizer, AstraZeneca, MSD and GlaxoSmithKline.
- Honoraria and research funding from Sanofi.
- Honoraria, consulting/advisory role and research funding from Boehringer Ingelheim.
- Honorarium, consulting/advisory role and meeting support from Eisai.
- Research funding from Amgen, Lilly, Taiho Pharmaceutical, Mylan, Merrimack, Merck, Abbvie, Astellas Pharma, Biomarin, Daiichi Sankyo, Abraxis BioScience, AB Science, Asana Biosciences, Medivation, Exelixis, ImClone Systems, LEO Pharma, Millennium, inVentiv Health Clinical, Celgene, Covance, Janssen, Atlantis Clinical, INC Research and Halozyme.
- Research funding, consulting/advisory role and meeting support from Bristol-Myers Squibb.
- Consulting/advisory role and meeting support from Libbs
- Steering committee member for the IMpassion130 trial. He did not receive travel support or reimbursement as part of the activities for the IMpassion 130 steering committee.

Dr Reinert’s disclosures:

- Research funding from AstraZeneca.
- Speaker honoraria from AstraZeneca, Lilly, Novartis, Pierre Fabre, Pfizer.

Dr Werutsky has no disclosures of conflicts of interest.

## Figures and Tables

**Table 1.** Time to anti-HER2 regulatory approval in FDA and Brazil.

Drug/indication	FDA approval (year)	ANVISA approval (year)	SUS access authorization (year)
Trastuzumab/metastatic	1998	1999	2017
Trastuzumab/adjuvant	2005	2006	2012
Lapatinib/metastatic	2007	2007	Unavailable
Pertuzumab/metastatic	2012	2013	2017
Pertuzumab/neoadjuvant	2013	2016	Unavailable
Pertuzumab/adjuvant	2017	2018	Unavailable
TDM1 metastatic	2013	2014	Unavailable

Legend: FDA Food and drug Administration (US), ANVISA Agência Nacional de Vigilância Sanitária (Brazil), SUS Sistema Público de Saúde (Brazil).
